# Fabrication and characterization of electrospun poly(e-caprolactone) fibrous membrane with antibacterial functionality

**DOI:** 10.1098/rsos.160911

**Published:** 2017-02-08

**Authors:** Idris Cerkez, Ayse Sezer, Sukhwinder K. Bhullar

**Affiliations:** 1 Department of Fiber and Polymer Engineering, Bursa Technical University, Bursa, 16190, Turkey; 2 Department of Mechanical Engineering, Bursa Technical University, Bursa, 16190, Turkey

**Keywords:** electrospinning, membranes, biodegradable

## Abstract

This research study is mainly targeted on fabrication and characterization of antibacterial poly(e-caprolactone) (PCL) based fibrous membrane containing silver chloride particles. Micro/nano fibres were produced by electrospinning and characterized with TGA, DSC, SEM and mechanical analysis. It was found that addition of silver particles slightly reduced onset of thermal degradation and increased crystallization temperature of neat PCL. Silver-loaded samples exhibited higher tensile stress and lower strain revealing that the particles behaved as reinforcing agent. Moreover, addition of silver chloride resulted in beaded surface texture and formation of finer fibres as opposed to the neat. Antibacterial properties were tested against Gram-negative and Gram-positive bacteria and remarkable biocidal functionalities were obtained with about six logs reduction of *Staphylococcus aureus* and *Escherichia coli* O157:H7.

## 1. Introduction

Electrospun membrane structures have unique properties such as increased surface-area-to-volume ratio, which makes them a good candidate for diverse areas including medical, environmental, agricultural and energy sciences [1]. Applications of these structures specifically in tissue engineering and drug delivery have been growing for the past several years [2]. Biocompatible and biodegradable polymer nanofibres with sizes less than one micrometre are especially useful in the field of medicine, because these nanomaterials replicate components of *in vivo* cellular and molecular environment. Moreover, they are beneficial for burn and wound healing due to their large surface-area-to-volume ratio, high porosity, improved cell adherence, cellular proliferation and migration, and controlled *in vivo* biodegradation rates. The large surface area of polymer nanofibre mats not only allows increased close interaction of therapeutic agents with tissues but also provides a mechanism for sustained release and localized delivery of drug [2–5]. These fibrous membranes are fabricated from both synthetic and natural polymers using electrospinning [6–8].

Poly(e-caprolactone) (PCL) is a biocompatible and biodegradable polymer making it a good candidate for medical applications. A number of medical devices are composed of PCL and remarkable efficacy studies have been reported in the literature [9–13]. Electrospinning draws great attention due to simplicity, cost-effectiveness, production of very thin fibres with large surface area and possibility of large-scale production [14]. Electrospun PCL membranes find uses as wound dressings, filtration devices, tissue scaffolds, drug delivery materials and medical implants [15]. All of these aforementioned PCL applications require antibacterial functionality due to increased number of infectious diseases. In this regard, various antimicrobial agents such as chitosan [16–19], silver [20–22], quaternary ammonium compounds [23] and chlorhexidine [24] have been used to render PCL biocidal. Among these biocides, silver and silver-based compounds are favourable due to broad spectrum activity, cheapness and resistance to bacterial mutation [25]. On the other hand, silver has disadvantage of having low biocompatibility. As cytotoxicity of silver compounds is very much dependent on silver amount, it is very critical to use at minimum concentration providing adequate biocidal activities [26–28]. Silver can be found in different forms such as salts (AgCl and AgNO_3_), colloid or elemental nanoparticles. Along with particles size, the form of silver affects its antibacterial power. Tomsic reported that bacterial reduction with silver salt is much better than silver nanoparticles [29]. In that case, it might be possible to obtain antibacterial activities using silver salts at lower concentration compared with nanoparticles. However, it is seen that most of the studies regarding biocidal PCL fibres dealt with silver nanoparticles rather than silver salts. For instance, Nirmala *et al*. have developed PCL containing hydroxyapatite-silver composite nanofibres through an electrospinning process for bone regeneration applications [30]. Similarly, Dubey *et al*. have reported silver nanoparticle containing poly(ethylene oxide)-PCL composite nanofibre and they concluded that the developed membranes provided ideal surface roughness, wettability and antimicrobial activity for use as wound-dressing scaffolds [23]. PCL-based polyurethane nanofibres containing silver nanoparticles were also developed for antimicrobial nanofilter applications [30]. This study reports use of silver chloride dispersion rather than silver nanoparticles to impart antibacterial functionality to PCL fibrous membrane. In this regard, micro/nano fibrous PCL membranes incorporating different amounts of AgCl were successfully fabricated and tested against *Staphylococcus aureus* and *Escherichia coli* O157:H7.

## 2. Experimental

### 2.1. Material and instrumentation

All chemicals were purchased from Sigma-Aldrich and used without further purification unless otherwise stated. Silver chloride dispersion, iSys AG, with a silver concentration of 8.4 mg g^−1^ was used as silver source and this product was kindly donated by CHT/Bezema, Turkey.

An Inovenso Nanospinner24 was used to fabricate the fibrous membranes. A Shimadzu AGS-X universal tester was used for testing mechanical properties of the produced webs. Thermal analysis data were collected at a heating rate of 10°C min^−1^ under nitrogen atmosphere using a Perkin Elmer STA 600 TGA and DSC 8000. Surface morphology of the fibres was characterized by a Carl Zeiss Evo 40 scanning electron microscope.

### 2.2. Electrospinning

Required amount of PCL (1.6 g) was dissolved in 7/1 w/w chloroform/methanol mixture in order to obtain 10 wt% PCL solution. After stirring the solution for 24 h at room temperature 5, 10 and 15 wt% AgCl dispersion based on PCL amount was added to this solution and stirred for 1 h at room temperature. Electrospinning of the fibres was carried out at 30 kV voltage and 0.25 ml min^−1^ flow rate. Fibres were collected on a rotating drum at 100 r.p.m. covered with aluminium foil. The distance between the collector and needle was adjusted to be 10 cm. Ten millilitres of solution was completely used for each sample in order to spin same amount of fibres. Taking account that iSys AG contains 8400 ppm silver [31], the final silver amount incorporated in PCL was calculated to be 420, 840 and 1260 ppm and these samples were denoted as 5, 10 and 15 wt% AgCl-PCL, respectively.

### 2.3. Antibacterial testing

ASTM E2149-01 test method was employed to determine antimicrobial activities of the fabricated samples against *S. aureus* (ATCC 6538) and *E. coli* O157:H7 (ATCC 35218). In brief, known concentration of the bacteria solution was suspended in 10 ml of saline water containing 0.1 g of nanofibres in closed sterile glass jars. The jars were agitated with a shaker for 24 h. Then, 10-fold serial dilutions were prepared and plated on Muller-Hinton II agar plate. The viable bacterial colonies were enumerated after incubation at 37°C for 24 h. The samples containing no fibres and the samples containing PCL fibres without AgCl were used as controls and treated in the same manner.

## 3. Results and discussions

### 3.1. Thermal analysis

**Figure 1.**
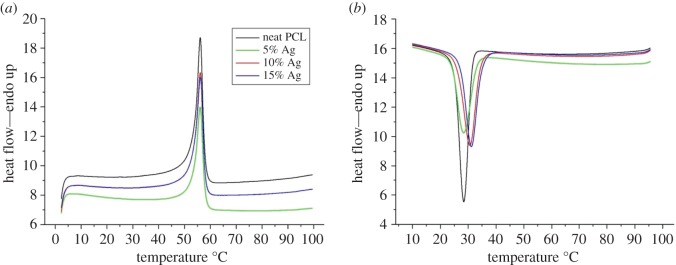
DSC thermograph of the produced fibres: (*a*) shows melting endotherm, (*b*) shows crystallization exotherm.

**Figure 2.**
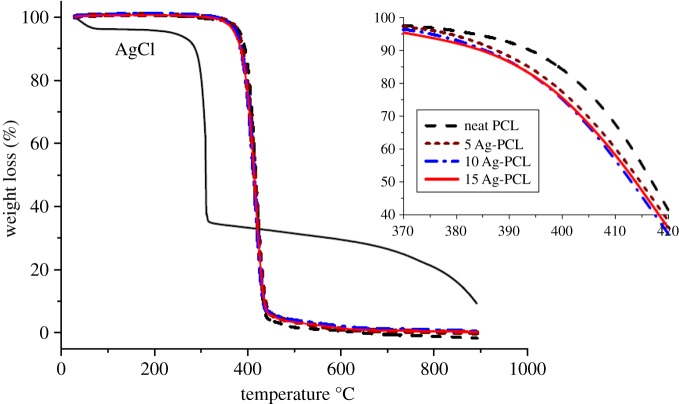
TGA thermograph of the produced fibres.

Figures 1 and 2 show DSC and TGA thermographs of the produced fibres. The neat PCL nanofibres showed melting and crystallization peaks at 55°C and 28°C, respectively. This is well consistent with the literature [32,33]. It was found that silver loading has not altered the melting temperature. On the other hand, incorporation of small amount of silver chloride slightly shifted crystallization temperature to a higher temperature. This result is due to silver chloride behaving as nucleation agent thus facilitating the crystallization process. However, not much difference in crystallization temperature was observed between the 10 and 15% AgCl containing fibres. This could be due to the agglomeration of the AgCl particles thus limiting the nucleating effect [34]. It is well known in the literature that polymers crystallize in two steps; nucleating and crystal growth. Even though homogeneous nucleation leads to better crystal formation, heterogonous nucleation is an easier and faster nuclei formation process than homogeneous nucleation. In this regard, it is believed that silver chloride particles facilitated the crystallization process by increasing the heterogonous nuclei formation caused by intermolecular interaction between the polymer chains and silver chloride. Nucleation effect of silver particles was also reported in other studies [35–37]. Finally, there was no significant change in heat of fusion of the samples signifying that silver loading did not alter the total crystallization amount of the PCL fibres.

As stated in other studies, it was found that thermal decomposition of silver chloride particles started at about 300°C along with a slight weight loss at temperature up to 100°C due to moisture evaporation [38]. The neat PCL nanofibres were found to be stable up to 380°C, whereas complete degradation took place at about 410°C [39,40]. Therefore, addition of silver chloride to fibre matrix was anticipated to lower thermal stability due to a two-stage thermal degradation process [41]. Even though the first stage, thermal degradation of the AgCl particles could not be significantly observed, earlier decomposition of the silver-chloride-containing fibres was obvious (figure 2 insert). On the other hand, not much difference was observed when silver amount was increased, due to relatively lower amount of silver chloride particles compared with the PCL matrix. In general, it can be concluded that addition of silver chloride did not significantly alter thermal decomposition pathway of the PCL fibres.

### 3.2. Surface characterization

**Figure 3.**
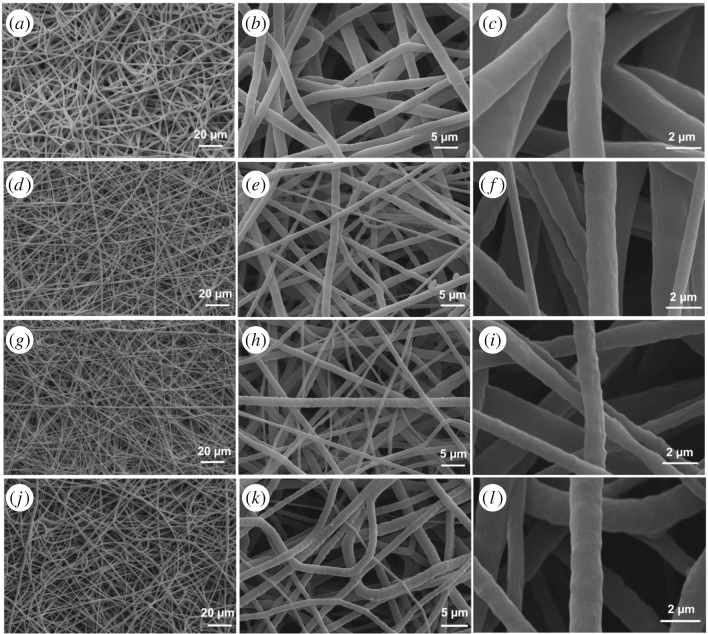
SEM images of neat PCL (*a*–*c*), 5 wt% AgCl-loaded PCL (*d*–*f*), 10 wt% AgCl-loaded PCL (*g*–*i*) and 15 wt% AgCl-loaded PCL (*j*–*l*).

Morphologies of the produced fibres were characterized by SEM analysis. As shown in figure 3, the neat PCL fibres had smooth surfaces and uniform fibre diameter distribution with an average fibre diameter of about 2 µm. Addition of silver chloride particles has led to finer fibre formations. In other words, micro/nano fibres were obtained together for the composite membranes. It is believed that this is related to solution electrical conductivity, as it is one of the critical parameters affecting the fibre diameter. There is a negative correlation between jet radius and conductivity. Addition of ionic salts enhances the charge-carrying capacity of the spinning solution resulting in finer fibre formation [34]. Therefore, it is speculated that addition of silver chloride increased the electrical conductivity of spinning solution, thus lowering the surface tension. Moreover, the beaded structure was observed on the surfaces of silver-containing fibres and this texture became more obvious with increasing amount of silver chloride. Beaded texture formation is believed to be due to agglomeration of the silver particles within the polymer matrices.

### 3.3. Mechanical testing

**Figure 4.**
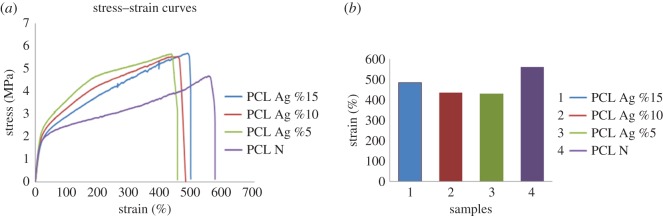
(*a*) Stress–strain curves of the membranes. (*b*) Maximum elongation bars.

A tensile test was performed to analyse mechanical behaviour of the membranes. The test was conducted at room temperature using 100 N load cell with a testing speed of 3 mm min^−1^ and a gauge length of 25 mm. As can be seen in the stress–strain curves shown in figure 4, addition of the silver chloride particles did not significantly affect the stiffness of neat PCL. On the other hand, silver loading dramatically increased the ultimate stress and slightly reduced strain at break. The neat PCL samples exhibited ultimate stress of about 4.6 MPa whereas about 5.7 MPa was obtained for PCL membranes containing 5% AgCl. This increment is due mainly to the presence of finer fibres in silver-chloride-loaded membranes as evidenced by SEM images in figure 3. The number of fibres present in membrane unit cross section is higher for silver-containing membranes which resulted in higher stress for breakage. Moreover, the physical interactions between the silver chloride particles and the polymer chains could also contribute to increased ultimate stress. It is speculated that ionic interactions between the positively charged silver atom and the partially negatively charged carbonyl groups of PCL resulted in higher intermolecular bonding between the polymer chains so that improved strength and reduced elongation at break were obtained [42]. Further increase in silver chloride amount slightly reduced ultimate stress compared with 5% AgCl samples. This resulted from non-uniform dispersion of AgCl particles within the PCL membranes leading to formation of defect points. Figure 3 clearly showed these agglomerated sections as beaded texture for 10 and 15% AgCl loaded fibres.

### 3.4. Antibacterial activities

**Figure 5.**
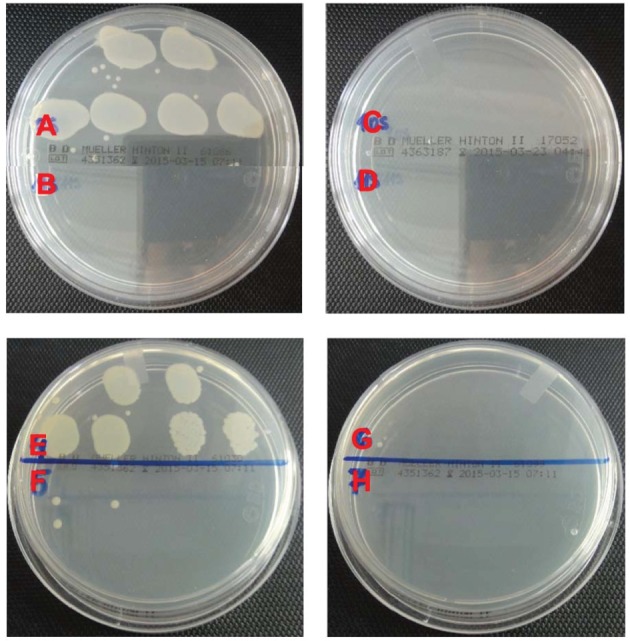
Biocidal test results of neat PCL (A and E), 5 wt% AgCl-loaded PCL (B and F), 10 wt% AgCl-loaded PCL (C and G) and 15 wt% AgCl-loaded PCL (D and H) against *S. aureus* and *E. coli* O157:H7, respectively.

Antibacterial activities of the samples were tested against Gram-positive and Gram-negative bacteria with *S. aureus* and *E. coli* O157:H7, respectively. About six logs of bacteria were challenged with the nanofibres according to ASTM E2149-01 test method. The results are shown in table 1. Blank sample without nanofibres (denoted as control in table 1) and neat PCL swatches both served as control samples. As can be seen, these control samples did not provide significant reduction. There was even an increase in the Gram-positive bacteria population for the control samples. The limited reduction that was observed for neat PCL swatches is believed to be due to adhesion of the bacteria to the fibre surfaces rather than inactivation [43]. On the other hand, silver-containing samples provided superior antibacterial functionalities with almost six logs reduction. The PCL swatches containing 5% silver chloride inactivated all of the Gram-positive bacteria, whereas 10% silver chloride was needed for total inactivation of the Gram-negative bacteria (figure 5). This is due to the extra lipid bilayer present in *E. coli* O157:H7 cell structure making these cells more resistant for silver penetration. In general, taking account that relatively small amount of silver was loaded in the PCL matrix, the biocidal results are found to be promising for use in various biomedical applications.

**Table 1.** Biocidal tests results.

	change in bacterial population (%)^a^	
samples	*S. aureus* (ATCC 6538)^b^	*E. coli* O157:H7 (ATCC 35218)^c^
control sample	(+) 180.7	(−) 55.6
neat PCL	(−) 22.8	(−) 27.3
5% AgCl-loaded PCL	(−) 100	(−) 99.2
10% AgCl-loaded PCL	(−) 100	(−) 100
15% AgCl-loaded PCL	(−) 100	(−) 100

^a^Positive sign indicates increment in bacterial population; negative sign indicates decrease in bacterial population.

^b^Initial bacterial concentration was 7.13 × 10^5^ (log 5.85) cfu.

^c^Initial bacterial concentration was 9.00 × 10^5^ (log 5.95) cfu.

## 4. Conclusion

Different amount of silver chloride particles were loaded in PCL and successfully formed into fibrous membrane using electrospinning. It was found that addition of silver particles did not alter melting temperature of the neat PCL, whereas a slight increase in crystallization temperature was obtained, as silver particles behaved as nucleation agent. TGA results revealed that silver-containing fibres slightly decomposed at earlier temperature and left higher char amount. Fibres about 2 µm were obtained when PCL was electrospun. On the other hand, finer fibre formation was observed for silver-containing fibres caused by increased solution electrical conductivity. Existence of finer fibres resulted in increased breaking strength and reduced extension. Surface morphology analysis showed beaded texture for the silver-loaded fibres, whereas smooth surfaces were obtained for the neat PCL. Finally, the silver-loaded fibres exhibited remarkable antibacterial functionalities such that about six logs of *S. aureus* and *E. coli* O157:H7 were inactivated, whereas no significant reduction was obtained for neat PCL.

In conclusion, silver-chloride-containing PCL fibrous membranes presented in this study possessed great potential for various biomedical applications including wound dressings, filtration, tissue scaffolds, drug delivery and medical implants and further tests such as biocompatibility and bio-absorbability need to be conducted.

## Supplementary Material

TGA raw data

## Supplementary Material

DSC raw data

## Data accessibility

The datasets supporting this article have been uploaded as part of the electronic supplementary material.
